# Supplementary material for: Connecting the Evolution and Spread of Turkey Reovirus Across the United States: A Genomic Perspective
Source: Viruses. 2025 Aug 29;17(9):1185. doi: 10.3390/v17091185 (PMC12474132; doi:10.3390/v17091185)
Supplement: Supplementary file 1 [file viruses-17-01185-s001.zip › Supplementary data.pdf]

Supplementary data

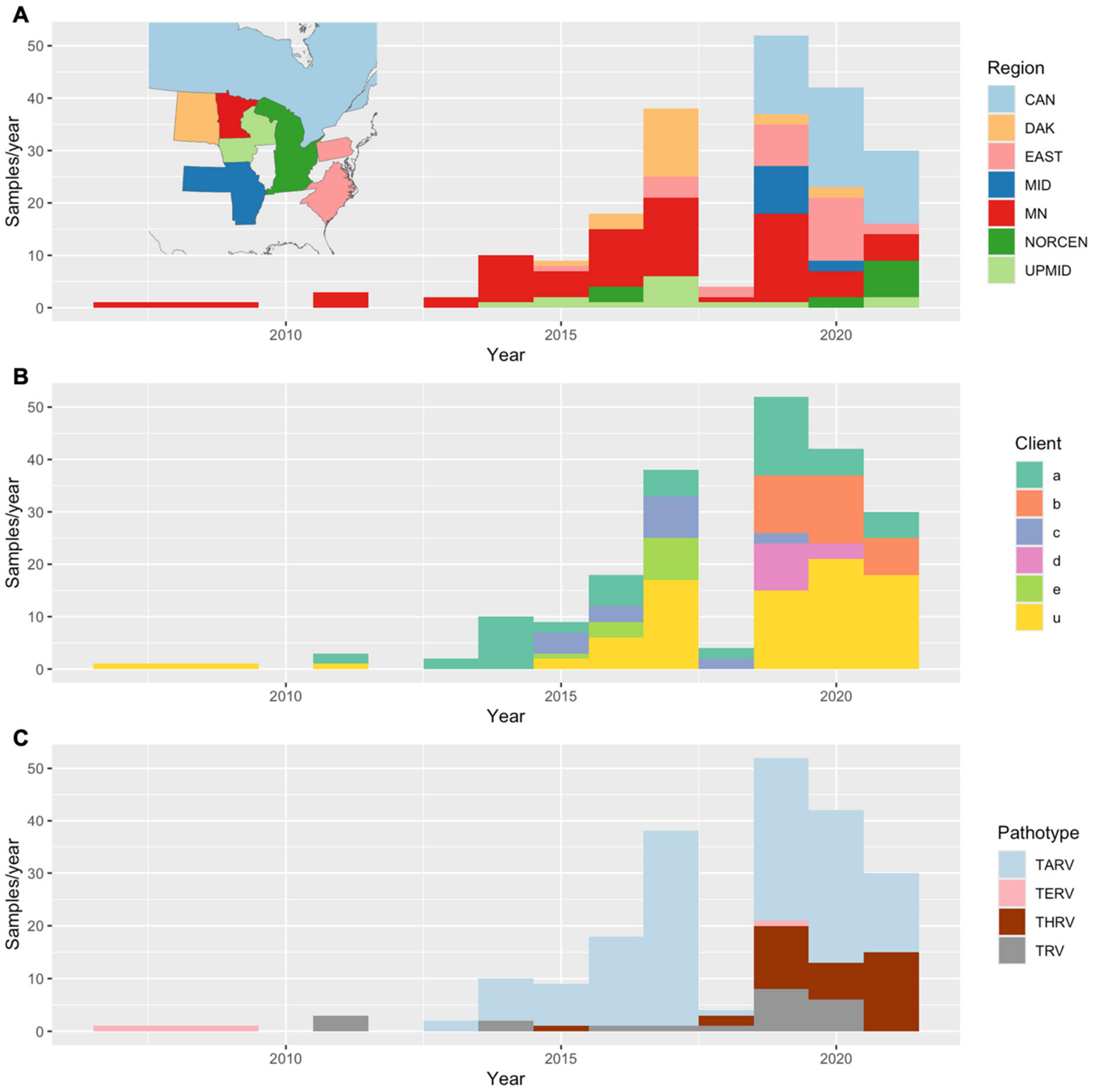

Supplementary Figure S1. Temporal distribution of the 211 TRV genome sequences analyzed in this study, showing the number of sequences per year. Bars are color-coded by key traits used in the phylodynamic analyses: geographic region (top), client group (middle), and pathotype classification (bottom).

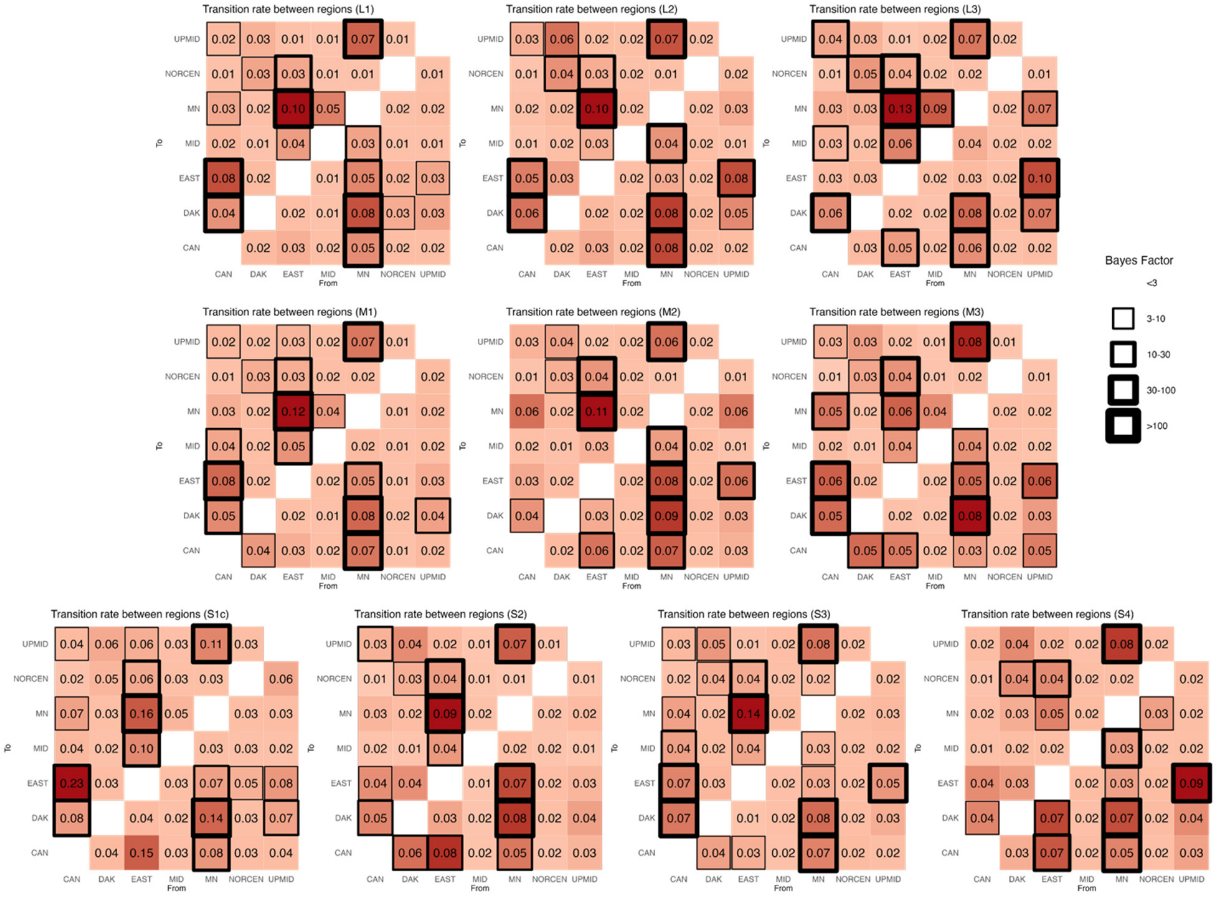

Supplementary Figure S2. Heatmaps illustrating the median transition rate between geographic regions based on phylodynamic analyses of ten TRV gene segments. Darker colors represent higher transition rate, while the thickness of cell borders reflects the strength of support, measured by the Bayes factor.

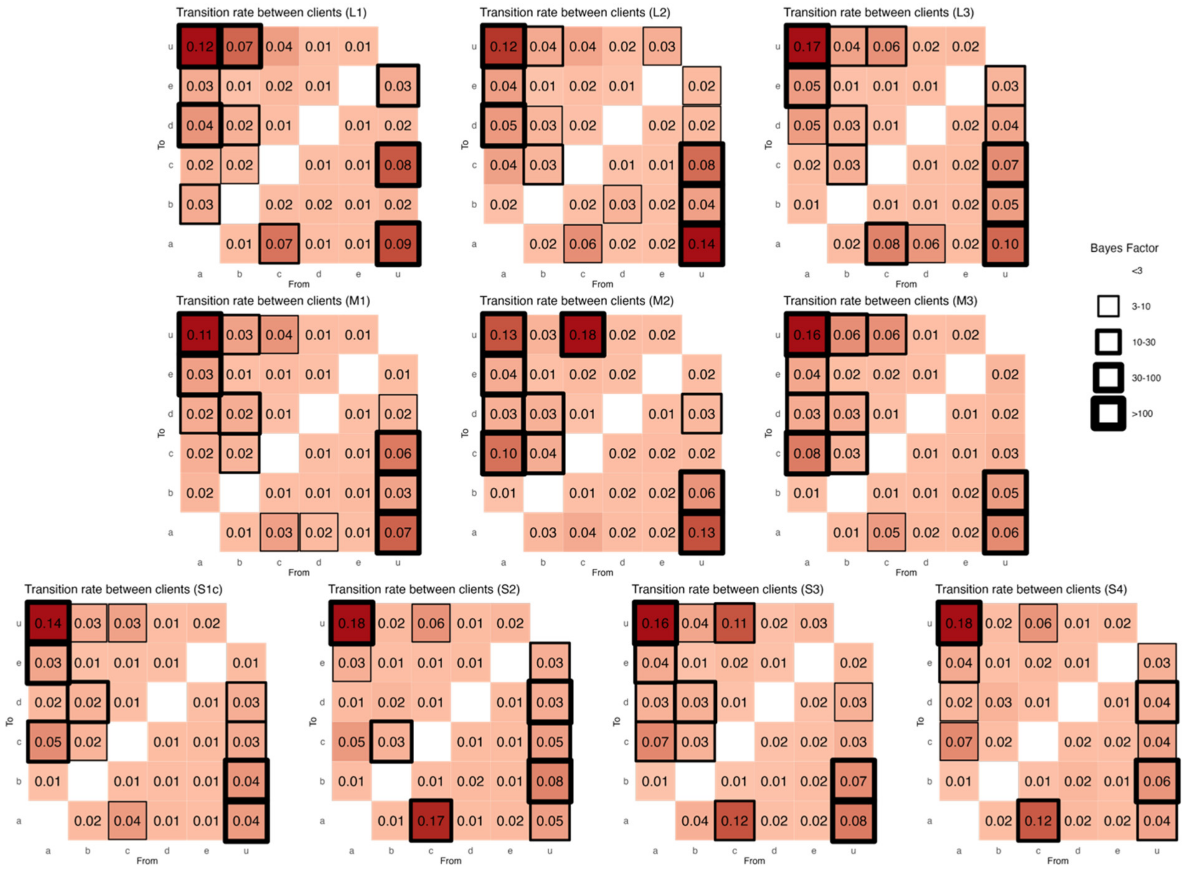

Supplementary Figure S3. Heatmaps illustrating the median transition rate between client groups based on phylodynamic analyses of ten TRV gene segments. Darker colors represent higher transition rate, while the thickness of cell borders reflects the strength of support, measured by the Bayes factor.

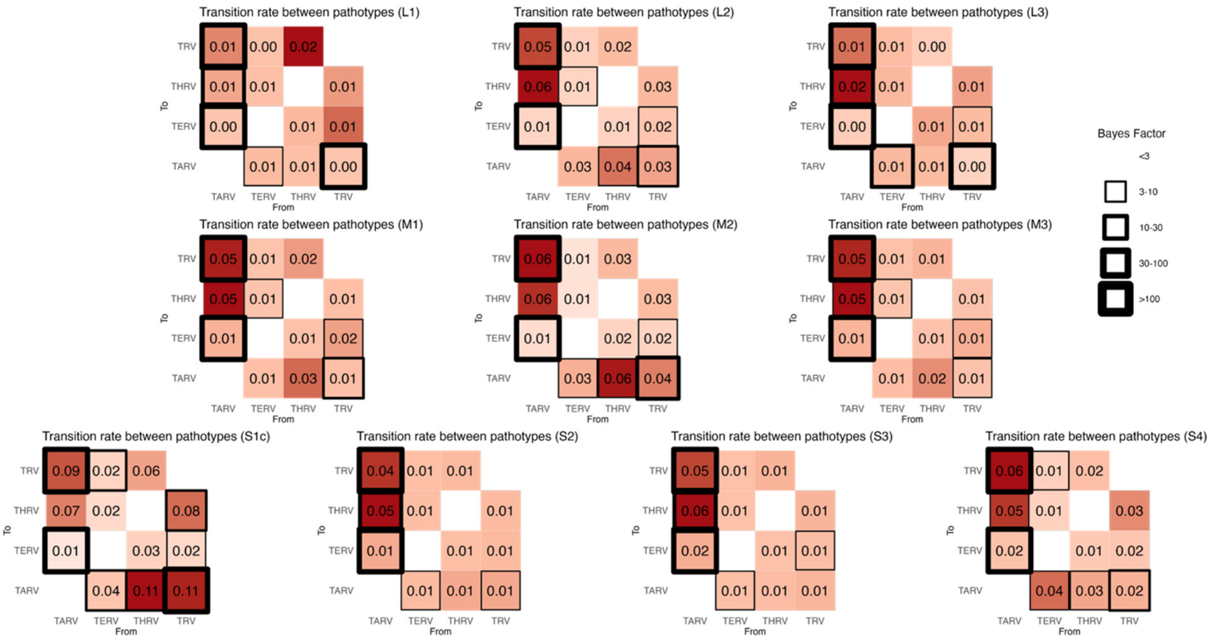

Supplementary Figure S4. Heatmaps illustrating the median transition rate between pathotypes (pathotype shift) based on phylodynamic analyses of ten TRV gene segments. Darker colors represent higher transition rate, while the thickness of cell borders reflects the strength of support, measured by the Bayes factor.

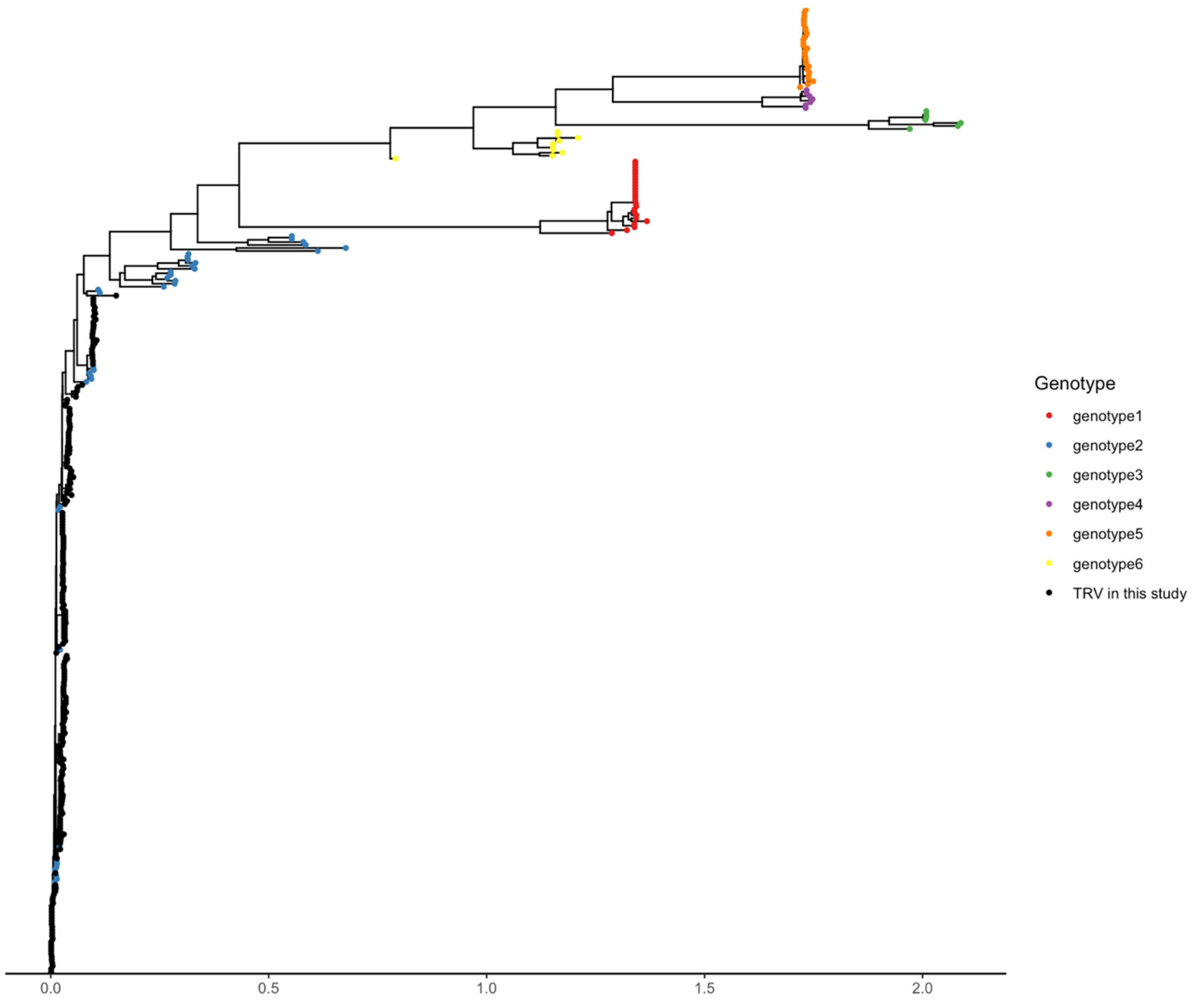

Supplementary Figure S5. Maximum likelihood phylogenetic tree of S1c gene segment of ARV samples used for genotyping in Lu et al., 2015 and TRV samples used in this study. Tip point colors represent genotypes classified in Lu et al., 2015.
